# Quantitative analysis of factors influencing tissue-engineered bone formation by detecting the expression levels of alkaline phosphatase and bone γ-carboxyglutamate protein 2

**DOI:** 10.3892/etm.2015.2259

**Published:** 2015-02-04

**Authors:** ZEZHONG SONG, CHANGSHUN WU, SHUI SUN, HUIBO LI, DONG WANG, JIANBAO GONG, ZEXING YAN

**Affiliations:** Joint Surgery Department, Shandong Provincial Hospital Affiliated to Shandong University, Jinan, Shandong 250021, P.R. China

**Keywords:** bone defects, β-tricalcium phosphate scaffold, bioreactor, platelet-rich plasma, rabbit bone marrow mesenchymal stem cells

## Abstract

Bone tissue engineering is a promising alternative approach that permits the efficient reconstruction of bone defects. There are four elements involved in bone tissue engineering technology, including the seed cells, growth factors, scaffolds and culture environment. The aim of the present study was to evaluate the effect of these factors on bone formation in tissue engineering technology by analyzing the expression of osteogenetic markers using polymerase chain reaction (PCR). Bone marrow mesenchymal stem cells (BMSCs) were extracted from the bone marrow of the bilateral tibial platform of New Zealand white rabbits. In addition, platelet-rich plasma (PRP) samples were prepared from blood extracted from the ear vein of the rabbits. A perfusion bioreactor was used to provide the culture environment, and β-tricalcium phosphate (β-TCP) was used to build the scaffolds. The β-TCP scaffolds were divided into five groups and each group was treated with a different combination of the factors. Next, the composites were implanted into the rabbits. After three months, the expression levels of the new bone formation markers, alkaline phosphatase and bone γ-carboxyglutamate protein 2, were detected using quantitative reverse transcription-PCR analysis. The expression levels of the markers in the experimental groups were higher compared with the negative control group. Comparisons between the experimental groups also revealed statistical significance. Scanning electron microscopy revealed good adhesion and distribution of the BMSCs on the β-TCP scaffold. In conclusion, the PCR results indicated that PRP, BMSCs and the bioreactor exhibited a promoting effect on bone formation.

## Introduction

Large bone defects constitute a major challenge for reconstructive surgery. Currently, clinically established therapeutic approaches for critically-sized bone defects mainly include autografts, allografts and artificial materials alone or in combination with bone grafts (1). However, a limited source of graft material is available for autogenous bone grafting, resulting in surgical morbidity of the donor site. In addition, allografts pose a risk of immunogenic reactions, disease transmission and inflammation. In recent years, the treatment of bone defects using tissue engineering technology has attracted increasing attention (2). The key elements of tissue engineering technology are the seed cells, growth factors and scaffolds. In addition, the culture environment of the tissue-engineered bones plays a significant role. Bone marrow mesenchymal stem cells (BMSCs) are multipotent cells that are able to differentiate into osteoblasts, adipocytes, tenocytes and marrow stroma.

BMSCs can be extracted from bone marrow and rapidly expanded *in vitro*; therefore, the cells have been widely used as seed cells (3). Platelet-rich plasma (PRP) is rich in growth factors that influence bone regeneration. When activated by an agonist, such as thrombin, PRP releases factors, including platelet-derived growth factor, epidermal growth factor, basic fibroblast growth factor, fibronectin and insulin-like growth factor-I. These factors have been demonstrated to be effective in the osteogenetic induction of BMSCs (4,5). Scaffolds play a critical role in the practical realization of bone tissue engineering. Various three-dimensional (3D) scaffolds are available, including ceramics, synthetic β-tricalcium phosphates (β-TCP) and calcium phosphates extracted from corals. In the present study, β-TCP was selected due to its high biocompatibility, good biological absorbability and ability to induce spontaneous osteoblastic differentiation and amplification (6). A perfusion bioreactor was used to simulate *in vivo* conditions and create a 3D environment, promoting cell adhesion, proliferation and differentiation. The effect of the bioreactor used in the present study was also assessed.

## Materials and methods

### Animal model and protocol

In total, 10 adult male New Zealand white rabbits (age, 2–3 months; weight, 1.7–2.3 kg) obtained from the Experimental Animal Center of Shandong Province (Jinan, China) were used in the study. All animal experimental protocols were approved by the Institutional Animal Care and Use Committee of Shandong University (Jinan, China), complying with the ‘Guide for the Care and Use of Laboratory Animals’ published by the National Academy Press (NIH publication no. 85-23, revised 1996). The animals were housed in separate cages at an ambient temperature of 24°C, and followed a standard diet. The condition, activities and excretion of the rabbits were monitored daily. Food and water were withheld at 6 and 1 h prior to surgery, respectively.

### 3D scaffold

A porous bioceramic 3D scaffold consisting of β-TCP (Shanghai Bio-lu Biomaterials Co., Ltd., Shanghai, China) was used. The β-TCP scaffold had an irregular cellular structure which provided a high porosity. The scaffold porosity was 75±10%, and >80% of the pores were spherical with a diameter of 500–600 μm. The acquired mechanical strength of the scaffold was high due to the spherical pores and smooth walls. The surface pores were continuous with the external environment and were connected to adjacent pores. A cylinder with a diameter and height of 5 mm was constructed using a mold. The cylindrical surface was smooth and was sterilized using ethylene oxide (7).

### Isolation and cultivation of rabbit BMSCs

The rabbits were anesthetized with 3% pentobarbital sodium (1 ml/kg) injected into the ear vein. With the rabbits under anesthesia, bone marrow aspirate (5 ml) was aspirated through the tibial tuberosity using a sterile bone marrow aspiration needle containing 1 ml heparin. The bone marrow was mixed with 1 ml sodium citrate (5%) prior to placing in an ice tray. BMSCs were isolated using the Percoll separation method. After mixing with an equal volume of D-Hank’s solution (Gibco^®^, Invirogen Life Technologies, Carlsbad, CA, USA) and homogenizing, the aspirate solution was centrifuged at 1,000 × g for 6 min. The supernatant was discarded and the remaining solution was mixed and homogenized with an equal volume of D-Hank’s solution. Next, an equal volume of Percoll separating medium (Solarbio Science & Technology Co., Ltd., Beijing, China) was added to the sample. Following centrifugation at 2,500 × g for 20 min, the cloudy solution in the middle of the centrifuge tube was harvested. After the addition of 5 ml D-Hank’s solution to the centrifuge tube, the sample was centrifuged at 1,000 × g for 6 min. Next, the BMSCs were harvested from the bottom of the centrifuge tube, and subsequently cultured in Dulbecco’s modified Eagle’s medium (Gibco^®^, Invitrogen Life Technologies), containing 10% fetal bovine serum (Gibco^®^ Life Technologies), and identified using flow cytometry (BD Biosciences, Frankin Lakes, NJ, USA) with CD34-fluorescein isothiocyanate (FITC) and CD44-FITC purchased from eBioscience Inc. (San Diego, CA, USA). Previous studies revealed that BMSCs are positive for CD44 and negative for CD34 (8,9). Cell growth was observed under an inverted phase-contrast microscope (CKX31; Olympus Corporation, Tokyo, Japan). After three passages, the BMSCs were used to build cell scaffold composites.

### Perfusion bioreactor

**Figure 1:**
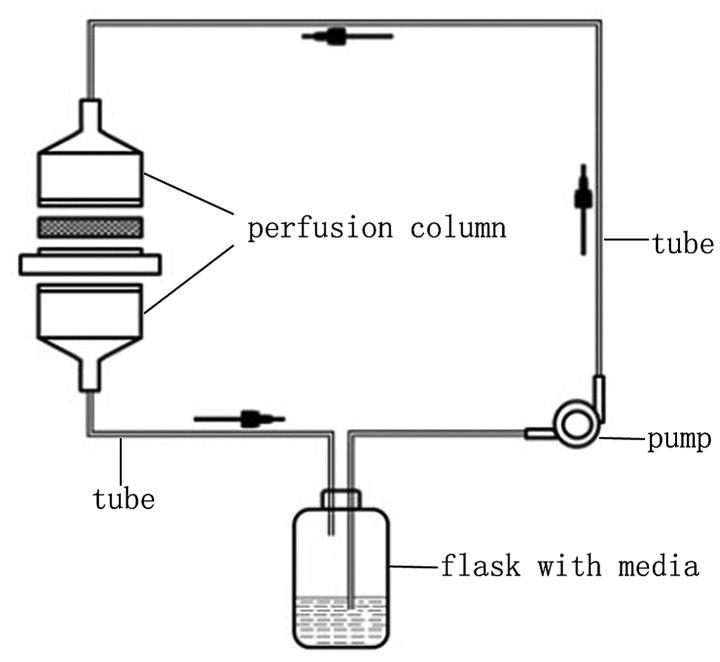
Schematic diagram of the perfusion bioreactor.

The perfusion bioreactor (Fig. 1) was designed at the East China University of Science and Technology (Shanghai, China). The system consisted of a peristaltic pump (Masterflex peristaltic pump; Cole-Parmer, Vernon Hills, IL, USA), a perfusion column (including the ring and sample tank), an air filter, two silicone tubes, a 3D interlinked connector and cell culture media. Two silicone tubes were used to transport culture media between the perfusion column and the flask, creating a circulation that simulated the internal body environment (10).

### Preparation and evaluation of the PRP

PRP was prepared according to a two-step centrifugation method. While the animals were under general anesthesia, 5 ml blood was drawn from the central ear artery with a 10-ml sterilized syringe containing 1 ml sodium citrate, which was used as an anticoagulant. Following homogenization, the mixture was centrifuged at 3,500 × g for 6 min to separate the cells from the serum components. Subsequently, the plasma and buffy layers were collected and subjected to further centrifugation at 3,000 × g for 6 min. Following removal of the top layer, the lower part of the solution was resuspended and designated as PRP. Next, the PRP was mixed and homogenized with 1 ml coagulant, consisting of 1 ml CaCl_2_ (10%) and 1,000 units thrombin. PRP gel, which had a jelly-like composition, was subsequently harvested.

### Tissue engineering bone building

Third generation BMSCs were prepared into a suspension prior to addition into the β-TCP scaffold. Upon addition of the suspension, the bracket was turned upside down to ensure adhesion in all parts of the bracket surface. Next, 3 ml cell culture media was added to each cultivation orifice plate and the scaffold was placed in a 37°C and 5% CO_2_ incubator overnight. The constructed bracket composites were then placed in the bioreactor perfusion column and cultured for three weeks in culture media with or without PRP, depending on the group allocation. The entire system was placed in a 37°C and 5% CO_2_ incubator for these three weeks, maintaining the CO_2_ flow rate at 3.5 ml/min. The culture medium was changed every 2–3 days, when the glucose content of the medium was depleted. The adhesion, proliferation and growth of the BMSCs in the β-TCP scaffold were examined using scanning electron microscopy (SEM; JSM-T300; JEOL-Technics Co. Tokyo, Japan). The bracket composites were divided into five groups as follows: Group A, BMSCs cultured with PRP in the bioreactor; group B, BMSCs cultured with PRP without the use of the bioreactor; group C, BMSCs cultured in the bioreactor without PRP; group D, BMSCs cultured without PRP and the bioreactor; and group E, β-TCP scaffold only (used as a negative control).

### Composite implantation

Following anesthesia with pentobarbital sodium (1 ml/kg) injected to the ear vein, the rabbits were placed in a left lateral position with head and limbs fixed. The skin on the right side at waist level was longitudinally incised to expose the superficial fascia. The BMSC composites were implanted between the shallow and deep fascia under the right side of the rabbit’s waist skin, while the animals were under general anesthesia. Next, the composites were fixed with sterile sutures and their positions were recorded. In the present experiment, each group had 10 composites and they were planted into 10 rabbits one by one. There were five groups in total, so each rabbit was planted with five composites from five groups. The grouping of the transplants was similar to that of a previous study (10). The rabbits were humanly sacrificed three months following surgery, and the composites with the surrounding tissue cells were removed in order to perform quantitative reverse transcription-polymerase chain reaction (RT-PCR).

### Detecting the expression levels of alkaline phosphatase (ALP) and bone γ-carboxyglutamate protein 2 (BGLAP2) using quantitative RT-PCR

The primer sequences used in the quantitative RT-PCR for rabbit ALP were 5′-TGT GCGGGGTCAAGGCTAAC-3′ (forward) and 5′-GGCGTC CGAGTACCAGTTGC-3′ (reverse), while for rabbit BGLAP2, the primers used were 5′-CTCCTTACCCGGATCCCCTG-3′ (forward) and 5′-GTAGAAGCGCTGGTAGGCGT-3′ (reverse). Total RNA was isolated using TRIzol^®^ reagent (Ambion^®^, Invitrogen Life Technologies) according to the manufacturer’s instructions. The cDNA sample was generated using the RevertAid First Strand cDNA Synthesis kit (Thermo Scientific, Waltham, MA, USA). Quantitative RT-PCR was performed using SYBR Green Realtime PCR Master mix (Toyobo, Osaka, Japan) in the Applied Biosystems AB7500 real-time PCR system (Applied Biosystems^®^, Invitrogen Life Technologies). The amplification was carried out under the following conditions: 95°C for 60 sec followed by 40 cycles at 95°C for 15 sec and 60°C for 60 sec.

### Statistical analysis

Quantitative RT-PCR experiments were performed in triplicate. The data are presented as the mean ± standard deviation and were analyzed using SPSS 18.0 statistical software (SPSS, Inc., Chicago, IL, USA). One-way analysis of variance or t-tests were used to compare the differences between groups, where P<0.05 was considered to indicate a statistically significant difference.

## Results

### Cell culture observation

**Figure 2:**
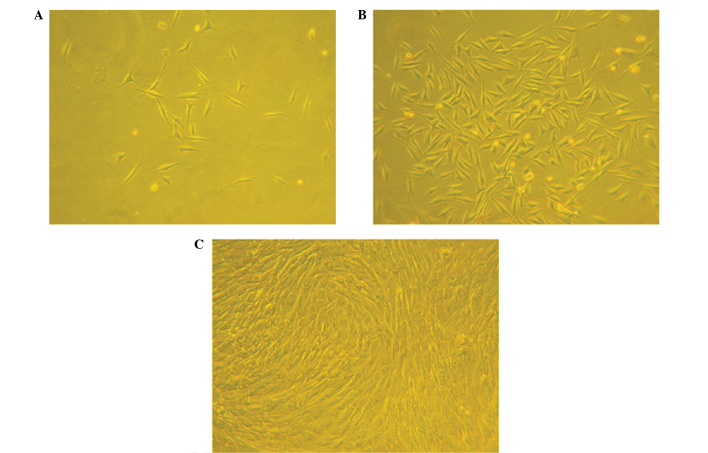
Bone marrow mesenchymal stem cells observed under an inverted phase-contrast microscope (magnification, ×100) at (A) day three following seeding, (B) day seven of culturing and (C) day seven following passage.

Observation of the BMSCs using an inverted phase-contrast microscope revealed a small amount of cell adhesion at day three following seeding. The cells were found to have a spindle-shape at this time. Cell colonies were formed after the primary cells had been cultured for seven days, and the cells were gathered into swirl-shaped colonies at day seven following passage (Fig. 2).

### Identification of BMSCs

Flow cytometric analysis of BMSCs was used to determine the gene expression profiles of cell passage 3. The results indicated that 2% of the cells were CD34-positive and 99.8% were CD44-positive.

### Composite examination with SEM

**Figure 3:**
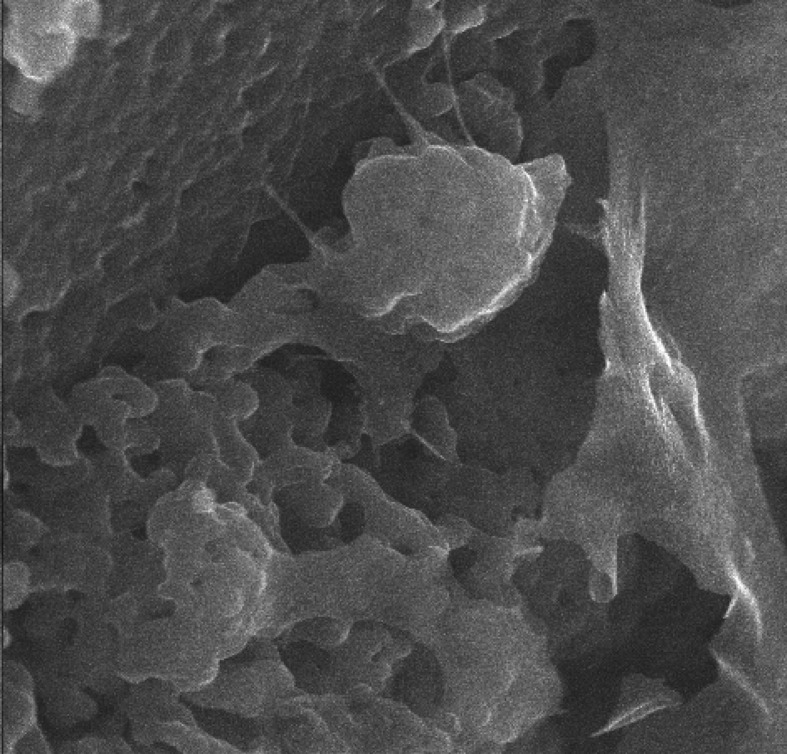
Scanning electron microscopy (magnification, 75 kv × 3 k) revealed good adhesion and distribution of bone marrow mesenchymal stem cells on the β-tricalcium phosphate scaffold.

SEM revealed good adhesion and distribution of BMSCs on the β-TCP scaffold. In addition, extracellular matrix secretion was observed on the scaffold. Favorable cell proliferation and stretch were also observed using SEM, indicating that the β-TCP scaffold had a good affinity for the BMSCs (Fig. 3).

### Expression levels of osteogenic differentiation markers

**Figure 4:**
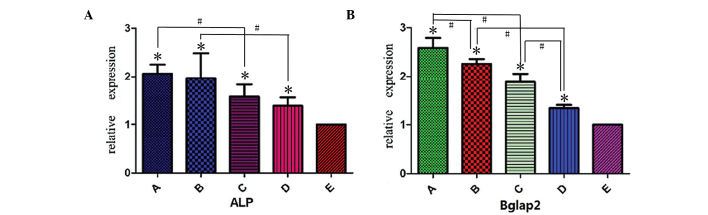
Gene expression levels of (A) ALP and (B) BGLAP2 were detected in the different study groups using quantitative reverse transcription-polymerase chain reaction. ALP, alkaline phosphatase; BGLAP2, bone γ-carboxyglutamate protein 2; group A, bone marrow mesenchymal stem cells (BMSCs) cultured with platelet-rich plasma (PRP) in the bioreactor; group B, BMSCs cultured with PRP without the use of the bioreactor; group C, BMSCs cultured in the bioreactor without PRP; group D, BMSCs cultured without PRP or the bioreactor; group E, β-tricalcium phosphate scaffold only. ^*^P<0.05 vs. control group (group E); ^#^P<0.05 vs. other experimental groups.

The expression levels of the osteogenic differentiation markers, ALP and BGLAP2, were detected using quantitative RT-PCR (Fig. 4). The expression levels of the markers were found to be much higher in the experimental groups (groups A-D) compared with the negative control group (group E; P<0.05). In addition, the expression levels of ALP and BGLAP2 were higher in group A compared with group C (P<0.05), and higher in group B compared with group D (P<0.05), indicating the promoting effect of PRP. In addition, the importance of BMSCs was demonstrated by the increased expression levels of the two markers in group D, as compared with group E (P<0.05). The expression level of BGLAP2 was found to be higher in groups A and C compared with groups B and D (P<0.05), respectively. Furthermore, the expression level of ALP was slightly higher in groups A and C compared with groups B and D, respectively; however, the differences were not found to be statistically significant (group A vs. B, t=0.177, P>0.05; group C vs. D, t=0.623, P>0.05). Therefore, the promoting effect of the perfusion bioreactor was demonstrated.

## Discussion

Research into the treatment of bone defects has always received much attention; however, in recent years, the rapid development of tissue engineering technology has provided a novel approach for the treatment of bone defects. The concept of tissue engineering involves the use of seed cells, growth factors and scaffolds (11). In addition, the culture of cells is essential in the development of tissue-engineered bones.

In the present study, bracket composites were constructed using BMSCs and a β-TCP scaffold. BMSCs were used as the seed cells, while PRP was used as a growth factor source. The composites were implanted into rabbits and the expression levels of bone formation markers were analyzed using quantitative RT-PCR. Alkaline phosphatase (ALP) and bone γ-carboxyglutamate protein 2 (BGLAP2) were used as indicators of cell osteogenesis differentiation. The activity of ALP is an important index for the evaluation of osteogenesis differentiation. In addition, ALP is an iconic enzyme of mature osteoblasts; thus, plays a critical role in the *in vitro* calcification process. Quantitative detection of the ALP concentration is frequently used for *in vitro* osteogenesis experiments as a conventional symbol of early osteoblast differentiation (12). BGLAP2 mainly appears in a mineralized formation of the cells as a sign of osteoblast maturation (13). Furthermore, the expression levels of ALP and BGLAP2 reflect the process of bone formation. As the quantitative RT-PCR results indicated, β-TCP scaffolds, BMSCs, PRP and the perfusion bioreactor had a significantly positive effect on bone formation.

BMSCs are derived from the mesoderm and ectoderm at an early developmental stage, and were first identified by Fridenshteĭn (14). BMSCs possess a multidirectional differentiation potential and can differentiate into osteoblasts, adipocytes, tenocytes and marrow stroma *in vivo* and *in vitro,* when induced under specific conditions. In addition, BMSCs preserve a multidirectional differentiation potential after continuous subculture and cryopreservation; thus, are ideal as seed cells (15).

PRP consists of concentrated platelets in a small volume of plasma. Upon activation by an agonist, the platelets in the PRP release the inflammatory and growth factors that are enclosed in their granules. PRP was first used in bone reconstruction therapy in the late 1990s; however, the role of PRP as a promoter of bone healing remains controversial (16). Previous studies have endorsed the ability of PRP to promote bone healing (17,18). By contrast, Kasten *et al* (19) investigated the effect of PRP on a critical-size diaphyseal radius defect in a rabbit model and observed that the combination of BMSCs and PRP had no additional effect on bone healing. However, the bone injury resulted from radial osteotomy, which may provide an explanation for the aforementioned observation, since growth factors and host precursors are known to be released following bone injury (20). The effect may have stimulated the BMSCs, not allowing a further increase due to PRP. In the present study, such influencing factors were excluded since the expression levels of new bone formation markers were detected using PCR, rather than observing the bone defect healing. Therefore, osteotomy was not performed and growth factors were not released due to bone injury. The data demonstrated that the addition of PRP promoted the bone formation process.

Scaffolds play a critical role in the practical realization of bone tissue engineering. In the current study, a porous bioceramic 3D scaffold made of β-TCP was used due to its favorable mechanical strength, high parity ratio, adjustable biodegradation rate and easy process and molding. β-TCP provides a 3D space, which is more conducive for cells to adhere, adapt to shear force and improve the unit expansion rate, promoting bone differentiation. In addition, β-TCP gradually releases bone induction growth factors in the process of cell culture due to the material characteristics (21). SEM demonstrated the good adhesion and distribution of BMSCs on the β-TCP scaffold, as well as extracellular matrix secretion. Favorable cell proliferation and stretch were also observed using SEM, indicating that the β-TCP scaffold has a good affinity for BMSCs.

Cell behavior is influenced by shear stress, tension stimulation and hydrostatic pressure stimulation. The perfusion bioreactor used in the present study applied continuous mechanical stimulation to the BMSCs with mediated fluid. In addition, the bioreactor provided the shear stress, tension and hydrostatic pressure stimulation required to simulate a stress environment *in vivo* (22,23). A previous study demonstrated that fluid shear stress has the most significant effect on cellular activity in the process of perfusion culture (24). Furthermore, the perfusion bioreactor system enhanced the ability to dynamically monitor stress. A 3D cultivation mode was established using the β-TCP scaffold and bioreactor, which exhibited a number of advantages over the traditional two-dimensional cultivation mode. The system improved the distribution of nutrients within the scaffold, while an *in vivo* environment was mimicked by providing mechanical stimulation with floating culture media. In addition, a previous study demonstrated that a contact inhibition effect appears when cells are in close contact at a certain stage of cell proliferation in the cell culture, which may inhibit the growth and proliferation of cells (25). Therefore, the 3D cultivation mode of the current study provided a 3D growth environment that promoted cell adhesion, growth and differentiation, reducing the inhibition of contact between the cells. The results of the present study demonstrated that the use of the bioreactor produced a better promotional effect on the expression of BGLAP2, as compared with ALP, during the osteogenetic differentiation process of the BMSCs, which should be addressed in future studies.

In summary, BMSCs, PRP, the β-TCP scaffold and the bioreactor had a positive effect on bone formation. BMSCs were found to be favorable seeding cells for tissue engineering. In addition, PRP was found to promote new bone formation through the combined action of the growth factors released, while the β-TCP scaffold was demonstrated to be suitable for BMSC adhesion and proliferation. Finally, the perfusion bioreactor provided a 3D culture mode that promoted cell adhesion, growth and differentiation; thus, it promoted bone formation. The construction of β-TCP scaffold composites using the 3D-bioreactor and PRP with BMSCs is an effective method of building tissue-engineered bones for bone formation. However, further comparative experiments are required to investigate the best choice of cells, growth factors, scaffolds and culture environment to build tissue-engineered bones.
